# Impact of age-related stereotype threat on subjective age, awareness of age-related change, and physical performance in older adults

**DOI:** 10.1007/s10433-025-00874-w

**Published:** 2025-08-25

**Authors:** Anna C. Müller, Anna E. Kornadt, Nanna Notthoff

**Affiliations:** 1 Faculty of Sport Science, Leipzig University, Leipzig, Germany; 2 Institute of Psychogerontology, Friedrich-Alexander-University of Erlangen-Nuremberg, Erlangen, Germany; 3 Department of Behavioural and Cognitive Sciences, University of Luxembourg, Esch-Sur-Alzette, Luxembourg

**Keywords:** Age-related stereotype threat, Age stereotypes, Subjective age, Awareness of age-related change, Physical performance

## Abstract

Age-related stereotype threat—the fear of being judged based on negative age stereotypes—can affect physical performance in older adults. This study postulated that stereotype threat influences sports motor performance and that self-perceptions of aging (SPA), specifically subjective age (SA) and awareness of age-related change (AARC), reinforce this relationship. A total of 86 adults aged 65 and over participated in the study (65–85; M = 72.10, SD = 5.94). Participants were randomly assigned to either an experimental group with stereotype threat or a control group. Physical performance was assessed using the advanced version of the sports motor test for adults. SPA (SA, AARC losses and gains) were measured by self-report. The results indicated that compared to the control situation, stereotype threat did not result in lower performance in individual sports motor domains (coordination, mobility, strength, endurance), but only in total performance. Greater AARC losses predicted lower performance in the strength domain. No moderating effect of SA was observed, but a moderating effect of AARC gains was identified in the coordination domain. Stereotype threat only led to lower performance in interactions with AARC gains and in tasks that required both cognitive and physical components. Our findings show that compared to SA, AARC appears more relevant to stereotype threat and physical performance. Finally, the impact of stereotype threat on motor performance is not universally detrimental, but rather domain-specific and only partially moderated by individual differences in SPA.

**Supplementary Information:**

The online version contains supplementary material available at 10.1007/s10433-025-00874-w.

## Introduction

Negative age stereotypes such as decline, frailty, and weakness are prevalent in Western societies and can significantly influence the behavior of older adults (Levy 2009; Levy and Myers 2004). For instance, negative age stereotypes can negatively influence engagement in physical activity and physical performance of older adults (Sarkisian et al. 2005; Emile et al. 2014; Swift et al. 2012). This is particularly important, as impaired physical motor skills can have a detrimental impact on health. For example, strength is linked to functional, psychological, and social health, balance performance predicts fall risk, and gait speed is a reliable indicator of functional status (Gale et al. 2007; Shumway-Cook et al. 2000; Middleton et al. 2016). The present study examines whether negative age stereotypes impact sports motor performance; self-perceptions of aging (SPA), including subjective age (SA) and awareness of age-related change (AARC), are considered as potential moderators.

### Stereotype threat

One explanation for the impact of negative stereotypes is *Stereotype Threat* (Steele and Aronson 1998). Stereotype threat is defined as a concern that occurs when individuals fear being validated or judged based on a negative stereotype, which can subsequently lead to impaired performance on tasks associated with the stereotype (Steele 1997). Studies have demonstrated that stereotype threat can impair physical performance in older adults. However, the number of studies investigating these effects remains limited, and the results are heterogeneous (e.g., Hausdorff et al. 1999; Horton et al. 2010; Swift et al. 2012; Barber et al. 2020), which warrants further study on potential moderating factors.

### SA and AARC as moderators

Existing research has shown that SPA, such as SA and AARC, are potentially relevant moderators in the relationship between stereotype threat and memory performance (Fernández-Ballesteros et al. 2015). SA is defined as the age people feel compared to their chronological age (Deeg et al. 2021; Kotter-Grühn et al. 2009), and laboratory studies have demonstrated a significant association between a higher SA and lower physical performance (Stephan et al. 2015; Ihira et al. 2015). Notthoff and colleagues (2018) observed this association exclusively in a laboratory setting (as measured by the Timed Up and Go Test [TUG]) and not in everyday life. This discrepancy may be attributed to the elicitation of stereotype threat in the laboratory setting, as the participants were under observation. The activation of stereotype threat may have impaired physical performance, with subjective age potentially moderating individuals’ susceptibility to this effect: feeling younger may buffer against performance decline, whereas feeling older may intensify it, consistent with previous findings (Fernández-Ballesteros et al. 2015).

SA is a rather broad indicator of individuals’ SPA. More recent work has attempted to understand the complex nature of SPA by considering contributions from different domains. Among these approaches is the concept of AARC, which encompasses all experiences that make a person aware of the changes in their performance and lifestyle due to aging. In contrast to the unidimensional concept of SA, AARC is a multidimensional concept that includes both positive and negative changes in various domains (Diehl and Wahl 2010). The results of previous studies have indicated that greater perceived AARC losses are associated with a slower walking speed (Barber et al. 2022).

SPA, including SA and AARC, arise from the internalization of age stereotypes over the life span (Levy et al. 2002a, 2002b). SPA can influence how older adults are affected by stereotype threat, with negative SPA exacerbating performance deficits and positive SPA acting as a protective factor (Fernández-Ballesteros et al. 2015). On the other hand, stereotype threat has also been found to modify SPA (Geraci et al. 2018; Eibach et al. 2010; Kotter-Grühn and Hess 2012). Therefore, SPA may plausibly serve as either moderators or mediators in the relationship between stereotype threat and relevant outcomes. The findings by Fernández-Ballesteros et al. (2015) and Notthoff et al. (2018) provide initial support for the hypothesis that SPA mainly influences the extent to which individuals are affected by stereotype threat, suggesting a moderating role. Nonetheless, the present study also investigates whether stereotype threat may alter SPA itself.

### Sports motor performance

To gain a comprehensive understanding of the impact of stereotype threat on physical performance, considering both SA and AARC, it is essential to examine various domains of sports motor performance. The study by Notthoff and colleagues (2018) revealed an association between SA and gait speed as assessed by TUG. However, performance in TUG is not solely dependent on walking speed; other physical factors, such as strength and mobility, also exert an influence (Benavent-Caballer et al. 2016). It is therefore necessary to investigate whether similar associations exist between SPA and other domains of sports motor performance. Notably, sports motor tasks must attain a certain level of difficulty to effectively reveal the impact of stereotype threat (Steele et al. 2002). This emphasis on task demands aligns with the biopsychosocial model (Lazarus & Folkman 1987), which posits that individuals’ reactions to performance situations depend on how they appraise their personal resources and the task’s demands.

### The present study

The objective of this study is to investigate the impact of stereotype threat on sports motor performance in different domains – coordination, mobility, strength and endurance. Furthermore, the study examines the role of SA (“How old do you feel?”) and AARC (losses and gains) in moderating the potential association. We hypothesize that older adults exposed to stereotype threat will exhibit poorer physical performance than older adults in the control condition (Swift et al. 2012; Barber et al. 2020). Moreover, it is assumed that the experience of stereotype threat will result in a higher SA, greater AARC losses and lower AARC gains (Eibach et al. 2010; Kotter-Grühn and Hess 2012; Wettstein et al. 2022). A higher SA, greater AARC losses and lower AARC gains may be associated with lower physical performance (Wang et al. 2022; Barber et al. 2022). Finally, it is hypothesized that a higher SA, greater AARC losses and lower AARC gains serve to reinforce the relationship between group assignment and performance, with the moderating effect being particularly pronounced in the experimental group. No specific hypotheses were formulated regarding the individual physical performance domains.

## Methods

### Participants

A priori power analysis using G*Power (Faul et al. 2007) indicated a total required sample size of 75 participants (*f* = 0.33; *α* = 0.05; (1- *β*) = 0.80), based on effect sizes reported in related studies (Swift et al. 2012; Lamont et al. 2015). To account for possible dropouts, a somewhat larger sample size was targeted, resulting in a final recruitment of 88 healthy older adults aged 65 and over in the area of Leipzig, Germany. Participants were recruited through advertisements or flyers distributed (e.g., in the local newspaper, health and rehabilitation centers, clubs). Data were collected between June and August 2023. Participants were screened with the Physical Activity Readiness Questionnaire (PAR-Q), in order to determine their suitability for physical activity (Krell-Rösch et al. 2014). After data collection, two participants had to be excluded from the analyses because they did not fully complete the sports test. Thus, a total of 86 test participants were included in the statistical analyses. 60.5% of the sample was female (Table 1), with an average age of 72 years (65–85;* M* = 72.10, *SD* = 5.93, Table 1).

**Table 1:** Chronological age and self-perceptions of aging of the sample by experimental group

	Total		Experimental group		Control group		Group differences	
	*M (Mz)*	*SD (SDz)*	*M1 (M1z)*	*SD1 (SD1z)*	*M2 (M2z)*	*SD2 (SD2z)*	*F*	*p-value*
Age	72.10	5.93	71.2	5.86	73.0	5.94	*F*(1,84) = 1.79	0.16
Subjective Age	-0.12	0.07	-0.11	0.08	-0.12	0.07	*F*(1,84) = 0.28	0.60
AARC Losses	10.02	2.86	10.30	3.24	9.77	2.45	*F*(1,84) = 0.69	0.41
AARC Gains	18.23	2.78	18.4	2.63	18.1	2.94	*F*(1,84) = 0.31	0.58

^*^Indicates *p* < 0.033, **indicates *p* < 0.001

### Procedure

The study was approved by Leipzig University’s ethics advisory board. The experiments were conducted by a 25-year-old woman with a Bachelor’s degree in sports science and an exercise instructor certification. The pre-test for assessing exercise ability using the PAR-Q test was conducted via telephone, email, or anonymously through the online survey platform Limesurvey. As part of this process, participants were also asked their age to ensure that asking this question on the test day would not induce stereotype threat (Shih et al. 1999). After giving written informed consent, older adults who met the inclusion criteria completed individual testing sessions that lasted approximately 60–90 min in a quiet laboratory. To anonymize data, participants drew lot numbers from 1 to 100. They were then randomly assigned to either the experimental group with stereotype threat or the control group. Both groups did not differ in terms of chronological age (*F*(1,84) = 1.94; *p* = 0.16, Table 1) and gender ($\chi^{2}$(1) = 0.07,* p* = 0.83, Table 2).

**Table 2:** Sample characteristics

	Total	Experimental group	Control group	Group difference
	n (%)	n (%)	n (%)	
Sex				$\chi^{2}$(1) = 0.07
Male	34 (39.5)	16 (38.1)	18 (40.9)	
Female	52 (60.5)	26 (61.9)	26 (59.1)	
Marital status				$\chi^{2}$(4) = 2.97
Married, living together	52 (60.5)	26 (61.9)	26 (59.1)	
Married, living separately	1 (1.2)	–	1 (2.3)	
Single	5 (5.8)	1 (2.4)	4 (9.1)	
Divorced	18 (20.9)	10 (23.8)	8 (18.2)	
Widowed	10 (11.6)	5 (11.9)	5 (11.4)	
School-leaving certificate				$\chi^{2}$(5) = 7.90
Junior high school	2 (2.3)	–	2 (4.5)	
Unified comprehensive 8/9th grade	4 (4.7)	3 (7.1)	1 (2.3)	
Secondary school	4 (4.7)	–	4 (9.1)	
Unified comprehensive 10th grade	20 (23.3)	11 (26.2)	9 (20.5)	
Vocational diploma	6 (7.0)	4 (9.5)	2 (4.5)	
Abitur	50 (58.1)	24 (57.1)	26 (59.1)	
Training qualification				$\chi^{2}$(12) = 14.44
None	1 (1,2)	–	1 (1.4)	
Apprenticeship	35 (40.7)	18 (29.0)	17 (24.6)	
Professionally qualified	5 (5.8)	4 (6.5)	1 (1.4)	
Public administration	2 (2.3)	–	2 (2.9)	
Public health	3 (3.5)	1 (1.6)	2 (2.9)	
Technical college DDR	12 (14.0)	2 (3.2)	10 (14.5)	
Master craftsman/technician	5 (5.8)	3 (4.8)	2 (2.9)	
Bachelor	3 (3.5)	2 (3.2)	1 (1.4)	
Diploma	41 (47.7)	21 (33.9)	20 (20.9)	
Master/ magister/ state Examination	7 (8.1)	3 (4.8)	4 (5.8)	
Doctorate	12 (14.0)	7 (11.3)	5 (7.2)	
Other degree	5 (5.8)	1 (1.6)	4 (5.8)	

^*^Indicates *p* < 0.033, **indicates *p* < 0.001

Participants assigned to the stereotype threat experimental group were informed that data would be collected regarding their performance in the sports motor test, attitude, experience, and socio-demographic background. It was emphasized that previous studies had identified age-related disparities in physical performance, attributing the diminished performance of older adults to factors such as coordination disorders, muscle atrophy, and the resultant heightened risk of falling, ultimately resulting in poorer performance compared to younger adults. Instructions were designed in alignment with Barber and colleagues (2020) and Brubaker and colleagues (2018), based on the Aronson and colleagues (1999) procedure (see Supplementary Information (SI)).

Participants assigned to the control group received instructions that also included preliminary information on data collection. They were told that the primary focus of the study was to investigate their individual physical performance on different tasks, that the sports test was feasible for all adult age groups, and that older adults with a health status similar to theirs performed well in sports motor tests. Homogeneity in readability of instructions for both groups was verified using the Flesch index (Flesch 1948).

Subsequently, participants completed measures on SPA. Participants then underwent the “advanced sports motor test” in the following order: coordination, mobility, strength, and endurance. The coordination, mobility, and strength tasks were administered in the laboratory; endurance data were collected outdoors on the test track of the Faculty of Sports Science at Leipzig University. All participants were able to complete the tests safely, and no adverse events occurred during the assessments.

After the sports motor test, we collected sociodemographic information. Finally, participants were debriefed on the exact aim of the study and the manipulation through stereotype threat. Participants received a five-euro drugstore (DM) voucher as compensation and a printout of their sports test results as feedback, given that no performance-related information was provided during the sports test (for the chronological procedure and an exemplar of the printouts, see SI).

### Measures

#### AARC

AARC was measured using the AARC SF-10 questionnaire (Kaspar et al. 2019), which includes five domains: health and physical function, cognitive function, interpersonal relationships, social-cognitive and social-emotional function, and lifestyle and engagement. Each behavioral domain assesses both losses and gains, with questions beginning with “As I get older, I notice that…”. Responses are given on a scale from 1 (not at all) to 5 (very much). The AARC-Losses and AARC-Gains scores were calculated based on the respective five items developed for both dimensions. For each scale, sum scores were computed, ranging from 5 (minimum) to 25 (maximum). Higher scores on the AARC-Gains scale indicate a greater perception of positive age-related changes, whereas higher scores on the AARC-Losses scale reflect a greater perception of negative age-related changes.

#### SA

SA was assessed by asking participants how old they feel in years. The question was integrated into the AARC questionnaire. Two versions of the AARC questionnaire varied the placement of the SA question—beginning or end—in order to minimize order effects. To calculate proportional discrepancy scores, the difference between SA and chronological age of each participant was divided by their chronological age (Stephan et al. 2013; Eibach et al. 2010; Rubin and Berntsen 2006). A positive score indicates that participants feel proportionately older than their chronological age, whereas a negative score indicates that they feel proportionately younger. Discrepancy values exceeding the sum of the third quartile and one and a half times the interquartile range or falling below the difference between the first quartile and one and a half times the interquartile range were replaced with values precisely corresponding to these thresholds. This method was selected for its capacity to objectively identify outliers, minimize bias from extreme values, and provide a reliable analysis of the data’s central tendency and variability (Bortz 2013).

#### Advanced version of the sports motor test for adults

Physical performance was assessed using the advanced version of the sports motor test for adults (Krell-Rösch et al. 2014). Sports motor tests examine the motor skills of coordination, mobility, strength and endurance, which form the basis for physical fitness and are of great importance for health and well-being (Krell-Rösch et al. 2014). Each sports motor domain in the advanced test is comprised of a distinct set of subtasks. In the present study, the advanced test was minimally modified by replacing “standing long jump” with “handgrip strength” due to the risk of injury for this age group. Grip strength has been implemented in other investigations examining stereotype threat in older adults (Horton et al. 2010; Swift et al. 2012). The advanced test provides standardized scores, with points scored as 0 “strongly below average”, 1 “below average”, 2 “average”, 3 “above average”, and 4 “strongly above average”. These values are based on reference values for the performance of different age groups and genders. In the domain of coordination, specific reference values were provided for 60+ year olds, and in the domain of endurance, reference values were provided for three older age groups: 60–69, 70–79, and 80–89 (Krell-Rösch et al. 2014). In contrast, only reference values up to the age of 59 were available for the domains of mobility and strength. Consequently, the reference values for the 60+ age group were calculated based on the average decrease across the age groups (see SI). The reference values for grip strength were derived from Steiber (2016) and classified according to the 0–4 point system (Steiber 2016; see SI). As the advanced test is aimed at fitness-oriented individuals, it is ensured that the test has a certain level of difficulty, which is a prerequisite for the activation of threat effects (Steele et al. 2002). An exercise ball, two exercise clubs, self-adhesive masking tape, a stopwatch, a measuring tape, a gymnastic stool with a measuring scale, an exercise stick with an attached centimeter scale, and an exercise mat were required to carry out the advanced test. Grip strength was measured using the HAMRY Model: EH101 dynamometer. All tasks were first demonstrated once by the experimenter.

The domain of coordination skills is comprised of two subdomains: the complex coordination test and the backward walking test. The complex coordination test is comprised of the following subtasks: hop run, ball grab, throwing with rotation, and figure-eight circle. Each task was attempted twice, with one point being awarded for each correctly solved attempt. A maximum of four points could be awarded in total. For the backward walking test, the distance (up to 6 m) traversed in a backward direction along a line was measured over time, with the best of three attempts being included. This value is then classified according to the reference Table (0–4 points). The coordination score was determined from the average of the two subdisciplines (0–4 points).

In the domain of mobility, two exercises are conducted: shouldering out and trunk flexion. In the trunk flexion test, participants stand on a stool with a measuring scale (in centimeters) attached to it. They then bend their trunk and move their hands as far down as possible. The better of the two attempts was considered and classified according to the reference Table (0–4 points). For shouldering out, participants moved a 1-m-wide measuring stick with outstretched hands first over their head and then behind their back. The width of the grip was constantly reduced until correct execution was no longer possible. Performance is calculated as the difference between participant’s grip width and their acromion distance. This value is then classified according to the reference Table (0–4 points). The mobility score was derived from the average of these two tasks (0–4 points).

The domain of strength included three tasks: grip strength, push-ups, and sit-ups. Two attempts were made for grip strength with the dominant hand, and the result of the better attempt was considered. This value was then classified according to the reference Table (0–4 points). Subsequently, the number of correctly performed push-ups within 40 s and then sit-ups within 30 s was counted and classified according to the reference Table (0–4 points). The strength score was calculated based on the average of the three sub-disciplines (0–4 points).

The endurance domain tests the aerobic endurance capacity and functional capacity of the leg muscles using a 2 km walking test. The test was carried out on the test track of the Faculty of Sport Science at Leipzig University, as poorer results are to be expected indoors (Krell-Rösch et al. 2014). Participants who began the test, but did not complete it were assigned 0 points. The duration of the 2 km walking test was classified according to the reference Table (0–4 points). The total score (max. 16) resulted from the sum of all four domains.

#### Covariates affecting sports motor performance

To test whether performance in the individual tasks is influenced by perceived exertion, participants were instructed to rate their perception of exertion immediately after completing each task. The modified Borg scale 0 to 10 was utilized to gauge the degree of perceived dyspnea, with ratings from 0 (no breathlessness) to 10 (maximum breathlessness) (Liu et al. 2020). Perceived difficulty was also assessed, as participants rated each task’s difficulty on a Likert scale from 1 (very easy) to 5 (very difficult) (Arnold et al. 1967). The individually perceived level of difficulty can predict the impact of the stereotype threat (Steele et al. 2002; Barber et al. 2020). Additionally, since the test took place in summer, and the endurance test was carried out outside, outside temperature was noted as a load parameter potentially impacting performance. Participants also reported their socio-demographic characteristics, including gender, age, marital status, educational level, and profession (Federal Statistical Office (Destatis) 2016). Chronological age and gender have been identified as factors in susceptibility to stereotype threat (Barber et al. 2017, 2020).

### Statistical analysis

R 4.4.3 and the packages dplyr, psych, car, ggplot2and emmeans were used for data analyses and illustration (Jockers et al. 2020; Revelle 2011; Fox et al. 2012; Wickham 2011; Lenth and Lenth 2018). Except for group and gender, all predictor variables were standardized. A general linear model with hierarchical structure was used to examine the effects of stereotype threat on physical performance in the domains of coordination, mobility, strength, endurance and total performance. Three different models were run for each domain. Model 1 included the experimental group (stereotype threat vs. control group), as well as SA, AARC losses and gains as predictors of physical performance. In line with Notthoff and colleagues (2018), a second model further included the stress parameters and demographic characteristics as covariates. Finally, Model 3 introduced the moderator terms of SA and AARC with group assignment. The interactions between perceived difficulty, chronological age, and gender with group assignment were also incorporated (Barber et al. 2017, 2020). To fully understand the combined impact on physical performance, a thorough examination of these interaction effects is required. To further probe the impact of the interaction variables that had a significant effect on physical performance, we ran separate regressions. The formulated directional hypotheses were tested one-tailed (α = 0.10). In order to control for type one error, a Bonferroni correction with α = 0.033 was applied. The calculation was based on three comparisons: (1) the control group, (2) the experimental group, and (3) the full sample (due to predictors applicable to the entire sample). Accordingly, the Bonferroni correction was adjusted using α divided by 3 (α/3).

## Results

### Stereotype threat and physical performance

The individual tasks did not reveal any significant differences between the two groups (Table 3). Compared to the control group, the experimental group had a lower overall average in all sports motor domains, with a statistically significant difference in total performance (*M*_EG_ = 8.45, *SD*_EG_ = 3.34, *M*_CG_ = 10.10, *SD*_CG_ = 2.64, *F*(1,84) = 5.46,* p* = 0.02,* η*^2^ = 0.06, 95% CI [0.00, 1.00], Table 3). When the predictors SA, AARC losses and gains, as well as covariates were incorporated into Models 1–3, the experimental group did not exhibit lower performance than the control group in any of the sports motor performance domains (*p* > 0.033) (Table 4).

**Table 3:** Scores, exertion, and difficulty in the coordination domain by experimental group

	Total		Experimental group		Control group		Group differences	
	M (Mz)	SD (SDz)	M1 (M1z)	SD1 (SD1z)	M2 (M2z)	SD2 (SD2z)	*F*	*p*-value
Coordination score	2.76	0.97	2.62	1.09	2.90	0.83	*F*(1,84) = 1.80	0.18
Difficulty	2.72	0.90	2.73	0.96	2.70	0.85	*F*(1,84) = 0.01	0.91
Exertion	0.31	0.43	0.31	0.41	0.30	0.46	*F*(1,84) = 0.004	0.95
Mobility score	2.30	1.38	2.10	1.32	2.50	1.43	F(1,84) = 1.86	0.18
Difficulty	2.74	1.21	2.81	1.21	2.68	1.23	F(1,84) = 0.24	0.63
Exertion	0.00	0.00	0.00	0.00	0.00	0.00	–	–
Strength score	2.45	0.83	2.37	0.86	2.52	0.79	F(1,84) = 0.78	0.38
Difficulty	2.28	1.12	2.46	1.16	2.10	1.06	F(1,84) = 2.28	0.13
Exertion	1.57	1.05	1.55	1.05	1.59	1.05	F(1,84) = 0.03	0.86
Endurance score	1.81	1.53	1.46	1.63	2.14	1.36	F(1,84) = 4.33	0.04
Difficulty	1.70	1.20	1.61	0.97	1.57	0.96	F(1,84) = 0.04	0.85
Exertion	2.26	1.69	2.27	1.62	2.18	1.59	F(1,84) = 0.07	0.79
Total score	9.32	3.08	8.54	3.34	10.1	2.64	F(1,84) = 5.46	0.02*
Difficulty	2.33	0.61	2.40	0.68	2.26	0.53	F(1,84) = 1.09	0.30
Exertion	1.02	0.62	1.03	0.62	1.02	0.63	F(1,84) = 0.01	0.93

^*^Indicates *p* < 0.033, **indicates *p* < 0.001

**Table 4:** Regression results of the hierarchical general linear models for investigating the association between group assignment, SA, AARC and sports motor performance

	Coordination score			Mobility score		
	M 1	M 2	M 3	M 1	M 2	M 3
Group	0.27 (*p* = 0.21)	0.30 (*p* = 0.09)	0.21 (*p* = 0.46)	0.28 (*p* = 0.21)	0.21 (*p* = 0.20)	− 0.33 (*p* = 0.24)
Subjective Age	− 0.01 (*p* = 0.92)	− 0.04 (*p* = 0.72)	− 0.06 (*p* = 0.66)	− 0.04 (*p* = 0.75)	− 0.05 (*p* = 0.58)	− 0.27 (*p* = 0.034)
AARC Losses	− 0.18 (*p* = 0.13)	− 0.05 (*p* = 0.64)	0.03 (*p* = 0.85)	0.02 (*p* = 0.89)	0.11 (*p* = 0.20)	0.16 (*p* = 0.17)
AARC Gains	0.12 (*p* = 0.25)	0.05 (*p* = 0.56)	− 0.23 (*p* = 0.11)	− 0.10 (*p* = 0.34)	− 0.06 (*p* = 0.43)	− 0.03 (*p* = 0.82)
Difficulty	–	− 0.59 (*p* < 0.001)**	− 0.82 (*p* < 0.001)**	–	− 0.62 (*p* < 0.001)**	− 0.79 (*p* < 0.001)**
Exertion	–	− 0.03 (*p* = 0.78)	− 0.07 (*p* = 0.47)	–	–	–
Temperature	–	0.08 (*p* = 0.41)	0.03 (*p* = 0.72)	–	− 0.16 (*p* = 0.05)	− 0.16 (*p* = 0.05)
Age	–	− 0.07 (*p* = 0.45)	− 0.11 (*p* = 0.46)	–	0.04 (*p* = 0.63)	− 0.06 (*p* = 0.63)
Gender	–	− 0.03 (*p* = 0.88)	− 0.17 (*p* = 0.57)	–	0.36 (*p* = 0.05)	− 0.24 (*p* = 0.41)
Group* Subjective Age	–	–	0.06 (*p* = 0.74)	–	–	0.30 (*p* = 0.10)
Group* AARC Losses	–	–	− 0.12 (*p* = 0.57)	–	–	0.02 (*p* = 0.90)
Group* AARC Gains	–	–	0.51 (*p* = 0.01)*	–	–	− 0.09 (*p* = 0.60)
Group* Difficulty	–	–	0.41 (*p* = 0.05)	–	–	0.23 (*p* = 0.19)
Group* Age	–	–	0.17 (*p* = 0.40)	–	–	0.11 (*p* = 0.51)
Group* Gender	–	–	0.10 (*p* = 0.79)	–	–	0.90 (*p* = 0.02)*
R^2^	0.07	0.43	0.53	0.03	0.53	0.58
F	1.64 (*p* = 0.17)	6.30 (*p* < 0.001)**	5.16 (*p* < 0.001)**	0.72 (*p* = 0.58)	10.76 (*p* < 0.001)**	7.14 (*p* < 0.001)**

	Strength score			Endurance score		
	M 1	M 2	M 3	M 1	M 2	M 3
Group	0.15 (*p* = 0.50)	− 0.02 (*p* = 0.88)	− 0.33 (*p* = 0.22)	0.41 (*p* = 0.06)	0.38 (*p* = 0.05)	− 0.08 (*p* = 0.82)
Subjective Age	0.06 (*p* = 0.59)	0.03 (*p* = 0.76)	0.02 (*p* = 0.90)	− 0.08 (*p* = 0.50)	− 0.20 (*p* = 0.07)	− 0.16 (*p* = 0.28)
AARC Losses	− 0.28 (*p* = 0.02)*	− 0.14 (*p* = 0.10)	− 0.17 (*p* = 0.13)	− 0.11 (*p* = 0.37)	− 0.05 (*p* = 0.61)	− 0.16 (*p* = 0.25)
AARC Gains	− 0.02 (*p* = 0.82)	0.02 (*p* = 0.82)	− 0.03 (*p* = 0.82)	− 0.01 (*p* = 0.93)	− 0.08 (*p* = 0.43)	− 0.08 (*p* = 0.64)
Difficulty	–	− 0.55 (*p* < 0.001)**	− 0.44 (*p* < 0.001)**	–	− 0.19 (*p* = 0.07)	− 0.21 (*p* = 0.17)
Exertion	–	0.39 (*p* < 0.001) **	0.40 (*p* < 0.001)**	–	0.23 (*p* = 0.04)	0.23 (*p* = 0.04)
Temperature	–	− 0.13 (*p* = 0.10)	− 0.10 (*p* = 0.20)	–	− 0.27 (*p* = 0.01)*	− 0.27 (*p* = 0.01)*
Age	–	− 0.05 (*p* = 0.58)	− 0.06 (*p* = 0.63)	–	− 0.13 (*p* = 0.20)	− 0.26 (*p* = 0.12)
Gender	–	0.10 (*p* = 0.57)	− 0.19 (*p* = 0.55)	–	− 0.73 (*p* < 0.001)**	− 1.13 (*p* = 0.002)*
Group* Subjective Age	–	–	− 0.06 (*p* = 0.72)	–	–	− 0.22 (*p* = 0.31)
Group* AARC Losses	–	–	0.12 (*p* = 0.53)	–	–	0.41 (*p* = 0.08)
Group* AARC Gains	–	–	0.04 (*p* = 0.82)	–	–	− 0.11 (*p* = 0.59)
Group* Difficulty	–	–	− 0.19 (*p* = 0.27)	–	–	0.07 (*p* = 0.72)
Group* Age	–	–	− 0.03 (*p* = 0.85)	–	–	0.12 (*p* = 0.57)
Group* Gender	–	–	0.53(*p* = 0.17)	–	–	0.79 (*p* = 0.08)
R^2^	0.07	0.62	0.63	0.07	0.33	0.39
F	1.64 (*p* = 0.17)	13.61 (*p* < 0.001)**	8.08 (*p* < 0.001)**	1.59 (*p* = 0.18)	4.18 (*p* < 0.001)**	3.04 (*p* < 0.001)**

	Total score		
	M 1	M 2	M 3
Group	0.45 (*p* = 0.04)	0.34 (*p* = 0.05)	− 0.08 (*p* = 0.77)
Subjective Age	− 0.04 (*p* = 0.71)	− 0.13 (*p* = 0.17)	− 0.17 (*p* = 0.19)
AARC Losses	− 0.18 (*p* = 0.13)	0.004 (*p* = 0.96)	− 0.07 (*p* = 0.59)
AARC Gains	− 0.02 (*p* = 0.85)	− 0.02 (*p* = 0.85)	− 0.10 (*p* = 0.51)
Difficulty	–	− 0.57 (*p* < 0.001)**	− 0.56 (*p* < 0.001)**
Exertion	–	0.28 (*p* = 0.003)*	0.27 (*p* = 0.01)*
Temperature	–	− 0.22 (*p* = 0.01)*	− 0.22 (*p* = 0.02)*
Age	–	− 0.07 (*p* = 0.45)	− 0.19 (*p* = 0.19)
Gender	–	− 0.05 (*p* = 0.80)	− 0.49 (*p* = 0.12)
Group* Subjective Age	–	–	− 0.02 (*p* = 0.94)
Group* AARC Losses	–	–	0.23 (*p* = 0.27)
Group* AARC Gains	–	–	0.06 (*p* = 0.76)
Group* Difficulty	–	–	0.07 (*p* = 0.72)
Group* Age	–	–	0.14 (*p* = 0.47)
Group* Gender	–	–	0.72 (*p* = 0.07)
R^2^	0.10	0.48	0.51
F	2.24 (*p* = 0.07)	7.67 (*p* < 0.001)**	4.87 (*p* < 0.001)**

^*^Indicates *p* < 0.033, **indicates *p* < 0.001

### SA and AARC as moderators

**Fig. 1:**
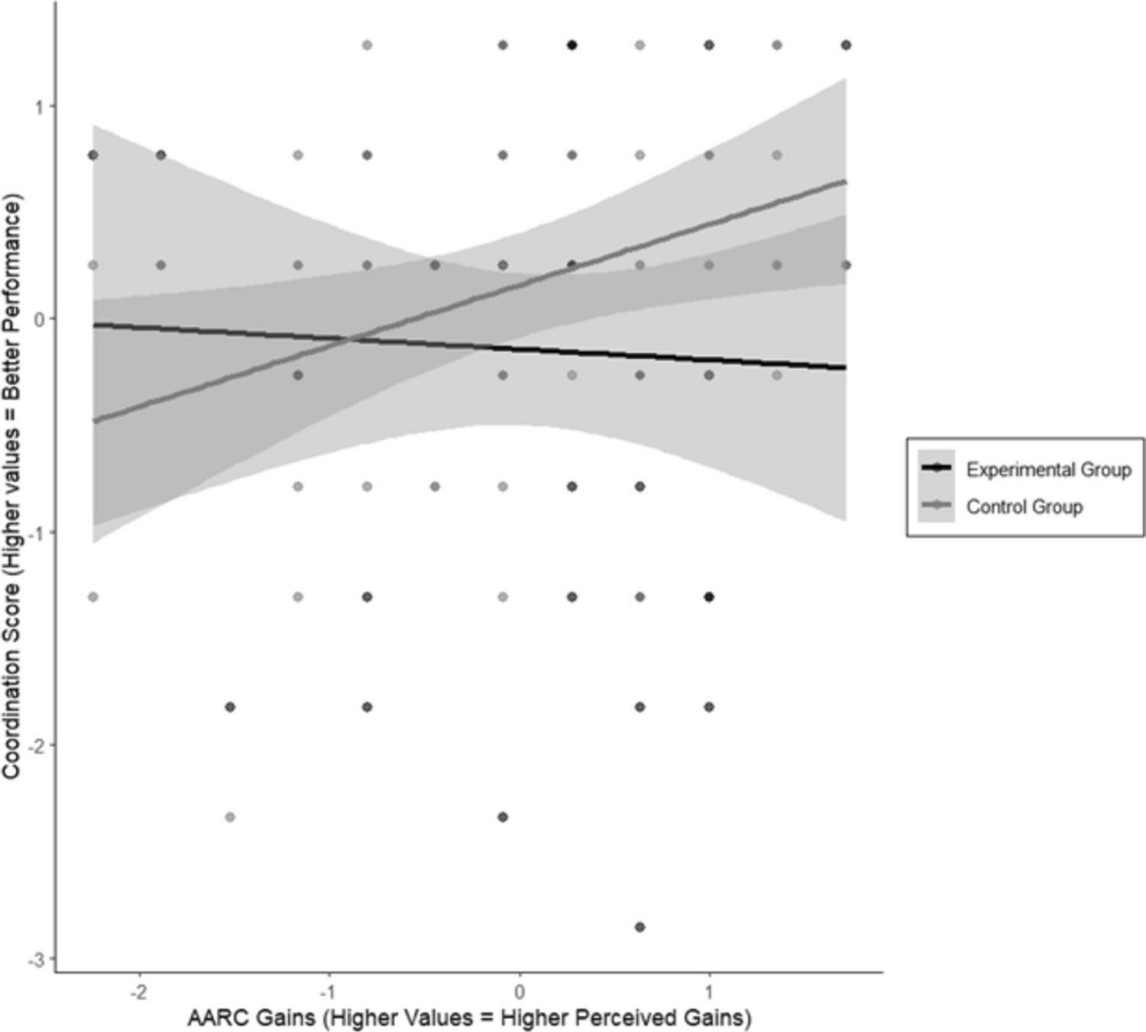
Graphical illustration of the association between the interaction of group assignment and AARC gains and coordination performance. The figure illustrates the moderation effect, showing that the groups respond differently to AARC gains in their performance

There were no significant differences between the groups in SA, AARC losses or AARC gains (*p* > 0.033, Table 1). In the domain of coordination, AARC gains in interaction with group assignment had a significant impact on performance in Model 3 (*b* = 0.51, *SE* = 0.18, *F*(1,70) = 7.69,* p* = 0. 0.01, *η*^2^ = 0.10, 95% CI [0.02, 1.00], Table 4, Fig. 1). A statistically significant difference in slopes was identified between the groups (*b* = − 0.51, *t*(70) = − 2.77, *p* = 0.01). Whereas the control group demonstrated significantly higher coordination performance with increasing AARC gains (*b* = 0.28, *t*(70) = 2.43, *p* = 0.02), the experimental group showed a non-significant trend toward lower performance (*b* = −0.23, *t*(70) = − 1.61,* p* = 0.11). A threshold analysis was conducted to determine the critical point at which the two groups significantly diverge. This analysis identified a value of 18.95 and above for AARC gains as the threshold at which the group difference became significant (*b* = − 0.40, *t*(70) = − 2.28, *p* = 0.03). In the strength domain, AARC losses were identified as a predictor of strength performance with greater AARC losses associated with lower performance (*b* = − 0.28, *SE* = 0.12, *F*(1,70) = 5.38,* p* = 0. 02, *η*^2^ = 0.06, 95% CI [0.01, 1.00]).

## Discussion

The aim of the present study was to examine whether negative age stereotypes impact sports motor performance; SA, AARC losses and gains were considered as potential moderators. The group exposed to stereotype threat exhibited a lower total performance; however, this effect was not sustained when controlling for additional variables and interaction terms. AARC gains were identified as a moderator in the association between group assignment and coordination performance. Furthermore, AARC losses were identified as a predictor of strength performance.

### Stereotype threat and physical performance

Our study goes beyond most previous studies that have failed to demonstrate effects of stereotype threat on physical performance by testing individual tasks (e.g., Hausdorff et al. 1999; Horton et al. 2010; Moriello et al. 2013). Our comprehensive battery of sports performance measures different domains and also allows for the calculation of a total score. Our findings that stereotype threat was associated with lower total performance suggests that the impact of stereotype threat may only become apparent in a cumulative manner. However, the findings were not robust when other variables, particularly task difficulty and effort, were included in the analysis. Except for endurance, task difficulty and effort were significantly related to physical performance in all domains, suggesting that stress parameters or task demands may have confounded the effects of threat on total performance.

The important role of task demands is not surprising, as theoretical models such as the biopsychosocial model suggest (Lazarus & Folkman 1987). In line with this rationale, we selected a demanding sports test to ensure that task demands were sufficiently high. However, the ratings indicated that the test was perceived as relatively easy, with no group difference. One possible explanation is that our scales did not assess participants’ perception of available resources, which may have varied between groups and could have influenced the persistence of stereotype threat effects (Barber et al. 2020).

### SA und AARC as moderators

In contrast to our expectations, the stereotype threat group did not report a higher SA, greater AARC losses and lower AARC gains compared to the control group. It is conceivable that the mere confrontation with stereotypes is not sufficient to change SPA; rather, manipulations may be more effective when the relevance of the stereotypes is experienced in the tasks performed. Besides, previous studies that have found an effect of stereotype threat on SPA have measured it after stereotype threat-related tasks (Eibach et al. 2010; Stephan et al. 2013). However, Fernández-Ballesteros et al. (2015) reported no significant differences in SPA among different experimental conditions, measured randomly before or after a memory test. These findings are consistent with the results of the present study and suggest that stereotype threat may not always manifest itself directly in SPA, but can still influence cognitive and behavioral outcomes (Steele and Aronson 1995).

We found no evidence for a direct effect of SA on performance, nor for a moderating effect. This finding was surprising and inconsistent with the results reported in previous studies (Stephan et al. 2015; Ihira et al. 2015). Still, AARC gains moderated the relationship between stereotype threat and coordination performance, when predictors, covariates, and interaction terms, were included. This suggests that stereotype threat may only manifest in those tasks that require the interaction of physical and cognitive systems (Golle et al. 2019). Coordination tasks demand situation-specific adaptations and adjustments and are not based solely on physical fitness levels as other domains. This characteristic may increase individuals’ susceptibility to stereotype threat, potentially explaining why previous studies mainly report declines in coordination performance (Barber et al. 2020; Borel et al. 2024; Chiviacowsky et al. 2018a). AARC losses were initially identified as a predictor in the strength domain, with greater perceived losses associated with lower performance. AARC is a pivotal indicator of physical functioning and health, with higher losses being associated with poorer physical health and reduced functional ability (Sabatini et al. 2020). Given the crucial role of muscle strength in physical functioning (Gale et al. 2007; Buchner & de Lateur 1991), it is reasonable that AARC losses would predict strength performance. The effect of AARC losses was overshadowed by perceived task difficulty and exertion.

In contrast to SA, AARC appears to be more pertinent to the relationship between stereotype threat and physical performance. This may be due to the fact that AARC reflects perceptions of age-related changes, providing a more direct link to internalized age stereotypes, which may amplify their effects (Rothermund et al. 2021; Brothers et al. 2017). Furthermore, multidimensional AARC has demonstrated a stronger association with health outcomes compared to unidimensional constructs (Sabatini et al. 2022).

### Strengths, limitations and future directions

Most previous studies examining the impact of stereotype threat on physical performance focused on individual tasks and found no effects. In addressing this restriction, the major strength of our study is that we used a comprehensive sports motor test rather than individual tasks, which allows conclusions to be drawn about specific domains as well as total performance. Another strength lies in the relatively large sample size per group compared to previous studies (e.g., Swift et al. 2012; Hausdorff et al. 1999; Horton et al. 2010).

In the present study, we examined the role of SA and AARC as moderators between group assignment and performance, as identified by Fernández-Ballesteros et al. (2015). However, only AARC gains moderated the impact of stereotype threat on coordination performance. Further studies should examine whether the observation that stereotype threat may only emerge in tasks that require the integration of physical and cognitive systems holds across similar task types. Additionally, subsequent research should investigate whether the timing of SPA assessment—whether it is administered before or after the performance test—affects the results.

Research that has reported significant effects (e.g., Barber et al. 2020; Borel et al. 2024) has primarily identified the effects of stereotype threat on difficult tasks. Although difficulty level has been demonstrated to influence performance outcomes in our study, it does not appear to amplify the effects of stereotype threat. This suggests that factors such as attitudes toward physical activity, resource evaluations, and self-efficacy may provide further insight (Moriello et al. 2013; Barber et al. 2020). A task can be perceived as difficult, but individuals may feel confident in their ability or resources to succeed. These factors may reduce the likelihood of stereotype threat, which should be included in future studies to better understand its impact on physical performance.

A key methodological limitation concerns the expected manipulation of stereotype threat, which did not lead to significant changes in SPA and sports performance, except for total performance. It is possible that the exposure to the manipulation alone is insufficient to trigger substantial alterations. Furthermore, the manipulation itself may not have been strong enough to elicit these effects. Despite following established methodologies, the instructions lacked a pretest to assess the efficacy of the experimental passages. Future research should explore different methods for testing manipulations, including pretesting or employing alternative approaches, to determine whether such manipulations can lead to significant changes in outcomes either independently or only after completing related tasks. A further limitation is related to the sports motor test used. Specifically, a calculation was made for the missing reference values for a few tasks (sit-ups, push-ups, shoulder extensions, and trunk flexions). Ideally, pre-evaluated reference values for the tests would have been utilized. Other sports motor tests with established reference values were not considered because they lack the requisite objective difficulty to elicit stereotype threat. Future studies should employ a variety of sports motor tests to ascertain whether the effects of stereotype threat, SA, and AARC remain consistent.

Based on previous research on stereotype threat in physical performance, our study was conducted as a single experiment within one country (e.g., Swift et al. 2012; Marquet et al. 2018; Barber et al. 2020). In order to enhance the generalizability of these findings, it is essential that future studies investigate stereotype threat effects across diverse cultural and national contexts. Furthermore, research suggests that stereotype threat effects may emerge over time. For instance, a study revealed significant effects only after a retention test (Chiviacowsky et al. 2018b). Since our study lacked a post-test follow-up, potential delayed effects should be investigated in future research. Additionally, our study differs from prior research by omitting several baseline variables (e.g., initial physical fitness, self-rated health, physical activity level, and anxiety) that did not show significant group differences (negative, positive, or control) in previous studies (Marquet et al. 2018; Barber et al. 2020). Instead, we focused on predictors, moderators, and covariates that have been identified as relevant in previous research. Excluding these variables may have limited our understanding of stereotype threat’s full impact. Although baseline health was screened using the PAR-Q to ensure suitability for physical activity, unaccounted differences could have influenced the results and should be addressed in future studies.

## Conclusion

The present study provides new insights into the role of stereotype threat in physical performance by considering a broad range of motor domains and examining the moderating effects of SA and AARC. Initial analyses suggested a negative impact, but this was not robust when additional variables were included. However, AARC gains moderated the relationship between stereotype threat and coordination performance, indicating that internalized SPA may influence responses to stereotype threat in physically and cognitively demanding tasks. Future research should retest sports motor tests and explore the role of SA and AARC, especially regarding the long-term effects of stereotype threat. A more thorough investigation could refine theoretical models and inform practical interventions in sports and rehabilitation contexts.

## Supplementary Information

Below is the link to the electronic supplementary material.

Supplementary file1 (DOCX 79 KB)

## Data Availability

No datasets were generated or analysed during the current study.
