# Supplementary material for: Impact of age-related stereotype threat on subjective age, awareness of age-related change, and physical performance in older adults
Source: Eur J Ageing. 2025 Aug 25;22(1):46. doi: 10.1007/s10433-025-00874-w (PMC12378272; doi:10.1007/s10433-025-00874-w)
Supplement: Supplementary file 1 — Supplementary file1 (DOCX 79 KB) [file 10433_2025_874_MOESM1_ESM.docx]

**Supplementary Information**

European Journal of Ageing: Social, Behavioural and Health Perspectives

Impact of age-related stereotype threat on subjective age, awareness of age-related change, and physical performance in older adults

Anna C. Schmidt^1,2^, Anna E. Kornadt^3^, Nanna Notthoff^1^

^1^Faculty of Sport Science, Leipzig University, Germany

^2^Institute of Psychogerontology, Friedrich-Alexander-University of Erlangen-Nuremberg, Germany

^3^Department of Behavioural and Cognitive Sciences, University of Luxembourg, Luxembourg

Author Note:

Anna C. Schmidt, ORCID: 0009-0005-2639-2086; Anna E. Kornadt, ORCID: 0000-0002-6634-0812; Nanna Notthoff, ORCID: 0000-0001-8707-2928; Corresponding author: Anna C. Schmidt, Email: anna.c.schmidt@fau.de

**Instructions for the Experimental and Control Conditions (Translated from German) (Adapted from Barber et al (2020) and Brubaker et al (2018))**

**Instruction of the experimental condition with stereotype threat**

Thank you very much for participating in this study. Today you will be performing sports motor tasks on coordination, mobility, strength and endurance. We will measure your performance on these tasks and collect data on your personal attitude and characteristics.

One of the main aims of this study is to assess age differences in the performance of the various sports motor skills and to investigate the mechanisms underlying the subtasks.

For this purpose, we are recruiting older adults aged 65 and over. It has been shown that age has an effect on physical performance. With increasing age, coordination disorders and muscle atrophy inevitably occur, which significantly increase the risk of falls in older adults. As a result, older adults generally underperform in sports motor tests compared to younger adults.

**Instruction of the control condition**

Thank you very much for participating in this study. Today you will be performing sports motor tasks on coordination, mobility, strength and endurance. We will measure your performance on these tasks and collect data on your personal attitude and characteristics.

One of the main aims of this study is to assess individual performance of the various sports motor skills and to investigate the mechanisms underlying the subtasks. In order to reduce possible age-related biases, a sports motor test is used that provides different standard values for all adult age groups. The test is feasible for all adult age groups.

For this purpose, we are recruiting older adults aged 65 and over. It has been shown that older adults with your state of health perform quite well in sports motor tests.

Table S1. Reference values for walking backwards

| Age | Sex | Points | | | | |
| --- | --- | --- | --- | --- | --- | --- |
|  |  | *4* | *3* | *2* | *1* | *0* |
| 30–39 | Female | >0,43 | 0,39–0,43 | 0,35–0,38 | 0,29–0,34 | <0,28 |
|  | Male | >0,50 | 0,45–0,50 | 0,41–0,44 | 0,36–0,40 | <0,35 |
| 40–49 | Female | >0,43 | 0,36–0,43 | 0,33–0,35 | 0,29–0,32 | <0,28 |
|  | Male | >0,50 | 0,44–0,50 | 0,39–0,43 | 0,34–0,38 | <0,33 |
| 50–59 | Female | >0,39 | 0,34–0,39 | 0,30–0,33 | 0,25–0,29 | <0,24 |
|  | Male | >0,49 | 0,43–0,49 | 0,38–0,42 | 0,33–0,37 | <0,32 |
| >60 | Female | >0,29 | 0,26–0,29 | 0,24–0,25 | 0,20–0,23 | <0,19 |
|  | Male | >0,38 | 0,36–0,38 | 0,32–0,35 | 0,28–0,31 | <0,27 |

*from Krell-Rösch et al (2014)

Table S2. Reference values for trunk bends

| Age | Sex | Points | | | | |
| --- | --- | --- | --- | --- | --- | --- |
|  |  | *4* | *3* | *2* | *1* | *0* |
| 20–29 | Female | >13 | 8–13 | 4–7 | -1–3 | <-1 |
|  | Male | >8 | 3–8 | -1–2 | (-7)–(-2) | <-7 |
| 30–39 | Female | >11 | 6–11 | 2–5 | -3–1 | <-3 |
|  | Male | >6 | 1–6 | -3–0 | (-9)–(-4) | <-9 |
| 40–49 | Female | >10 | 5–10 | 0–4 | (-4)–(-1) | <-4 |
|  | Male | >6 | 1–6 | -2–0 | (-9)–(-3) | <-9 |
| 50–59 | Female | >8 | 3–8 | 0–2 | (-5)–(-1) | <-5 |
|  | Male | >5 | 1–5 | -5–0 | (-11)–(-6) | <-11 |
| >60* | Female | >6 | 1–6 | -1–0 | -6–(-2) | >-6 |
|  | Male | >4 | 0–4 | -6–(-1) | -12–(-7) | >-12 |

*calculated using the average decrease across the age groups; adapted from Krell-Rösch et al (2014)

Table S3. Reference values for shouldering-out

| Age (in years) | Sex | Points | | | | |
| --- | --- | --- | --- | --- | --- | --- |
|  |  | *4* | *3* | *2* | *1* | *0* |
| 20–29 | Female | <42 | 42–51 | 52–59 | 60–70 | >70 |
|  | Male | <50 | 50–62 | 63–71 | 72–82 | >82 |
| 30–39 | Female | <46 | 46–55 | 56–63 | 64–74 | >74 |
|  | Male | <54 | 54–64 | 65–73 | 74–84 | >84 |
| 40–49 | Female | <53 | 53–60 | 61–68 | 69–78 | >78 |
|  | Male | <61 | 61–70 | 71–77 | 78–88 | >88 |
| 50–59 | Female | <56 | 56–66 | 67–73 | 74–84 | >84 |
|  | Male | <69 | 69–76 | 77–82 | 83–91 | >91 |
| >60* | Female | <61 | 61–71 | 72–78 | 79–89 | >89 |
|  | Male | <75 | 75–81 | 82–86 | 87–94 | >94 |

*calculated using the average decrease across the age groups; adapted from Krell-Rösch et al (2014)

Table S4. Reference values for grip strength

| Age (in years) | Sex | Points | | | | |
| --- | --- | --- | --- | --- | --- | --- |
|  |  | *4* | *3* | *2* | *1* | *0* |
| 65-69 | Female | >27,6 | 27,5–24,2 | 24,1–20,7 | 20,6–17,3 | <17,2 |
|  | Male | >44,1 | 44,0–39,3 | 39,2–34,4 | 34,3–29,6 | <29,5 |
| 70-74 | Female | >26,0 | 25,9–22,8 | 22,7–19,5 | 19,4–16,3 | <16,2 |
|  | Male | >41,7 | 41,6–36,7 | 36,6–31,6 | 31,5–26,6 | <26,5 |
| 75-79 | Female | >24,2 | 24,1–21,2 | 21,1–18,3 | 18,2–15,3 | <15,2 |
|  | Male | >37,8 | 37,7–32,6 | 32,5–27,3 | 27,2–22,1 | <22,0 |
| 80-90 | Female | >21,5 | 21,4–18,8 | 18,7–16,0 | 15,9–13,3 | <13,2 |
|  | Male | >33,2 | 33,1–28,2 | 28,1–23,3 | 23,2–18,3 | <18,2 |

*grip strength value below the mean value plus two standard deviations is declared weak (0 points) and a value above the mean value plus one standard deviation is declared strong (4 points) (Steiber, 2016)

Table S5. Reference values for push-ups

| Age (in years) | Sex | Points | | | | |
| --- | --- | --- | --- | --- | --- | --- |
|  |  | *4* | *3* | *2* | *1* | *0* |
| 20–29 | Female | >16 | 15–16 | 13–14 | 11–12 | <11 |
|  | Male | >22 | 21–22 | 18–20 | 16–17 | <16 |
| 30–39 | Female | >15 | 14–15 | 11–13 | 9–10 | <9 |
|  | Male | >20 | 19–20 | 17–18 | 15–16 | <15 |
| 40–49 | Female | >13 | 12–13 | 10–11 | 8–9 | <8 |
|  | Male | >15 | 14–15 | 11–13 | 9–10 | <9 |
| 50–59 | Female | >12 | 11–12 | 9–10 | 7–8 | <7 |
|  | Male | >14 | 13–14 | 10–12 | 8–9 | <8 |
| >60* | Female | >10 | 10 | 8–9 | 6–7 | <6 |
|  | Male | >12 | 11–12 | 7–10 | 5–6 | <5 |

*calculated using the average decrease across the age groups; adapted from Krell-Rösch et al (2014)

Table S6. Reference values for sit-ups

| Age (in years) | Sex | Points | | | | |
| --- | --- | --- | --- | --- | --- | --- |
|  |  | *4* | *3* | *2* | *1* | *0* |
| 20–29 | Female | >19 | 17–19 | 15–16 | 12–14 | <12 |
|  | Male | >22 | 19–22 | 16–18 | 13–15 | <13 |
| 30–39 | Female | >17 | 15–17 | 13–14 | 10–12 | <10 |
|  | Male | >21 | 17–20 | 14–16 | 11–13 | <12 |
| 40–49 | Female | >17 | 14–17 | 12–13 | 9–11 | <9 |
|  | Male | >19 | 16–18 | 14–15 | 12–13 | <11 |
| 50–59 | Female | >13 | 11–13 | 9–10 | 7–8 | <7 |
|  | Male | >18 | 15–17 | 13–14 | 10–12 | <10 |
| >60* | Female | >11 | 9–11 | 7–8 | 5–6 | <5 |
|  | Male | >17 | 14–17 | 12–13 | 8–11 | <8 |

*calculated using the average decrease across the age groups; adapted from Krell-Rösch et al (2014)

Table S7. Reference values for walking-test

| Age (in years) | Sex | Points | | | | |
| --- | --- | --- | --- | --- | --- | --- |
|  |  | *4* | *3* | *2* | *1* | *0* |
| 20–29 | Female | <14:19 | 14:19–15:24 | 15:25–16:20 | 16:21–17:25 | >17:25 |
|  | Male | <12:58 | 12:58–14:07 | 14:08–15:07 | 15:08–16:16 | >16:16 |
| 30–39 | Female | <14:53 | 14:53–15:58 | 15:59–16:54 | 16:55–17:59 | >17:59 |
|  | Male | <13:31 | 13:31–14:40 | 14:41–15:40 | 15:41–16:49 | >16:49 |
| 40–49 | Female | <15:27 | 15:27–16:32 | 16:33–17:28 | 17:29–18:33 | >18:33 |
|  | Male | <14:04 | 14:04–15:13 | 15:14–16:13 | 16:14–17:22 | >17:20 |
| 50–59 | Female | <16:01 | 16:01–17:06 | 17:07–18:02 | 18:03–19:07 | >19:07 |
|  | Male | <14:38 | 14:38–15:47 | 15:48–16:47 | 16:48–17:56 | >17:56 |
| 60–69 | Female | <16:35 | 16:35–17:40 | 17:41–18:36 | 18:37–19:41 | >19:41 |
|  | Male | <15:11 | 15:11–16:20 | 16:21–17:19 | 17:20–18:29 | >18:29 |
| 70–79 | Female | <17:09 | 17:09–18:14 | 18:15–19:10 | 19:11–20:15 | >20:15 |
|  | Male | <15:44 | 15:44–16:43 | 16:44–17:53 | 17:54–19:02 | >19:02 |
| Ab 80 | Female | <17:43 | 17:43–18:48 | 18:49–19:44 | 19:45–20:49 | >20:49 |
|  | Male | <16:17 | 16:17–17:26 | 17:27–18:26 | 18:27–19:35 | >19:35 |

*from Krell-Rösch et al (2014)

Table S8. Scores, exertion, and difficulty of all subtasks by experimental group

|  | Total | | Experimental Group | | Control Group | | Group Differences | |
| --- | --- | --- | --- | --- | --- | --- | --- | --- |
|  | *M (Mz)* | *SD (SDz)* | *M1 (M1z)* | *SD1 (SD1z)* | *M2 (M2z)* | *SD2 (SD2z)* | *F* | *p-value* |
| Complex Coordination Score | 2.63 | 0.85 | 2.73 | 0.89 | 2.55 | 0.82 | *F*(1,84) = 0.97 | 0.33 |
| Difficulty | 2.81 | 0.76 | 2.81 | 0.77 | 2.82 | 0.77 | *F*(1,84) = 0.003 | 0.96 |
| Exertion | 0.23 | 0.32 | 0.26 | 0.32 | 0.21 | 0.33 | *F*(1,84) = 0.55 | 0.46 |
| Walking Backwards Score | 2.91 | 1.46 | 2.57 | 1.65 | 3.23 | 1.18 | *F*(1,84) = 4.51 | 0.04 |
| Difficulty | 2.62 | 1.40 | 2.64 | 1.45 | 2.59 | 1.37 | *F*(1,84) = 0.03 | 0.87 |
| Exertion | 0.31 | 0.48 | 0.32 | 0.49 | 0.29 | 0.49 | *F*(1,84) = 0.09 | 0.76 |
| Trunk Bends Score | 2.79 | 1.47 | 2.69 | 1.49 | 2.89 | 1.47 | *F*(1,84) = 0.38 | 0.54 |
| Difficulty | 1.63 | 1.01 | 1.70 | 1.04 | 1.57 | 0.98 | *F*(1,84) = 0.38 | 0.54 |
| Exertion | 0.00 | 0.00 | 0.00 | 0.00 | 0.00 | 0.00 | - | - |
| Shoulder Mobility Score | 1.74 | 1.98 | 1.40 | 1.91 | 2.07 | 2.00 | *F*(1,84) = 2.46 | 0.12 |
| Difficulty | 3.69 | 1.66 | 3.76 | 1.62 | 3.61 | 1.71 | *F*(1,84) = 0.17 | 0.68 |
| Exertion | 0.00 | 0.00 | 0.00 | 0.00 | 0.00 | 0.00 | - | - |
| Hand Grip Strength Score | 3.05 | 1.00 | 3.14 | 1.03 | 2.95 | 0.99 | *F*(1,84) = 0.75 | 0.39 |
| Difficulty | 1.00 | 0.00 | 1.00 | 0.00 | 1.00 | 0.00 | - | - |
|  | - |  |  |  |  |  |  |  |
| Exertion | 0.00 | 0.00 | 0.00 | 0.00 | 0.00 | 0.00 | - | - |
|  | - |  |  |  |  |  |  |  |
| Push-ups Score | 2.31 | 1.45 | 2.02 | 1.47 | 2.59 | 1.39 | *F*(1,84) = 3.38 | 0.07 |
| Difficulty | 2.49 | 1.69 | 2.74 | 1.74 | 2.25 | 1.63 | *F*(1,84) = 1.80 | 0.18 |
| Exertion | 2.38 | 1.61 | 2.30 | 1.55 | 2.47 | 1.67 | *F*(1,84) = 0.23 | 0.63 |
| Sit-ups Score | 1.85 | 1.52 | 1.69 | 1.55 | 2.00 | 1.49 | *F*(1,84) = 0.89 | 0.35 |
| Difficulty | 3.00 | 1.72 | 3.29 | 1.70 | 2.73 | 1.72 | *F*(1,84) = 2.30 | 0.13 |
| Exertion | 2.31 | 1.85 | 2.32 | 1.91 | 2.31 | 1.82 | *F*(1,84) = 0.001 | 0.97 |
| Walking Score | 1.81 | 1.53 | 1.46 | 1.63 | 2.14 | 1.36 | *F*(1,84) = 4.33 | 0.04 |
| Difficulty | 1.70 | 1.20 | 1.61 | 0.97 | 1.57 | 0.96 | *F*(1,84) = 0.04 | 0.85 |
| Exertion | 2.26 | 1.69 | 2.27 | 1.62 | 2.18 | 1.59 | *F*(1,84) = 0.07 | 0.79 |

*Indicates *p* < 0.033, **indicates *p* < 0.001

Table S9. Separate regression results for the interaction between AARC gains and coordination performance by experimental group

| Total | Experimental Group | Control Group |
| --- | --- | --- |
| -0.51 | -0.23 | 0.28 |
| *t*(70) = -2.77 | *t*(70) = -1.61 | *t*(70) = 2.43 |
| *p* = 0.01* | *p* = 0.11 | *p* = 0.02* |

*Indicates *p* < 0.033, **indicates *p* < 0.001

Table S10. Separate regression results at the threshold of significant group difference for AARC gains (= 18.95)

| Total | Experimental Group | Control Group |
| --- | --- | --- |
| -0.40 | -0.18 | 0.22 |
| *t*(70) = -2.28 | *t*(70) = 1.47 | *t*(70) = 1.76 |
| *p* = 0.03* | *p* = 0.15 | *p* = 0.08 |

*Indicates *p* < 0.033, **indicates *p* < 0.001

Table S11. Correlation matrix of self-perceptions of aging by experimental group

| Experimental Group (Control Group) | (2) | (3) |
| --- | --- | --- |
| (1) Subjective Age | 0.39 (0.42) | 0.04 (0.04) |
| (2) AARC Losses | - | -0.33 (0.17) |
| (3) AARC Gains | - | 1 |

Table S12. Variance inflation factors (VIF) of predictors in the hierarchical general linear models

|  | Coordination | | | Mobility | | | Strength | | | Endurance | | | Total | | |
| --- | --- | --- | --- | --- | --- | --- | --- | --- | --- | --- | --- | --- | --- | --- | --- |
|  | *M1* | *M2* | *M3* | *M1* | *M2* | *M3* | *M1* | *M2* | *M3* | *M1* | *M2* | *M3* | *M1* | *M2* | *M3* |
| Group | 1.01 | 1.05 | 3.07 | 1.01 | 1.05 | 3.29 | 1.01 | 1.09 | 3.50 | 1.01 | 1.04 | 3.09 | 1.01 | 1.06 | 3.02 |
| Subjective Age | 1.21 | 1.31 | 2.61 | 1.21 | 1.27 | 2.68 | 1.21 | 1.31 | 2.72 | 1.21 | 1.33 | 2.52 | 1.26 | 1.33 | 2.51 |
| AARC Losses | 1.22 | 1.35 | 3.14 | 1.22 | 1.28 | 2.42 | 1.22 | 1.37 | 3.46 | 1.22 | 1.28 | 2.35 | 1.22 | 1.34 | 2.60 |
| AARC Gains | 1.02 | 1.07 | 3.04 | 1.02 | 1.06 | 3.07 | 1.02 | 1.09 | 3.09 | 1.02 | 1.07 | 3.18 | 1.02 | 1.06 | 3.10 |
| Group* Subjective Age |  |  | 2.47 |  |  | 2.63 |  |  | 2.61 |  |  | 2.50 |  |  | 2.59 |
| Group* AARC Losses |  |  | 2.57 |  |  | 2.22 |  |  | 2.44 |  |  | 2.20 |  |  | 2.31 |
| Group* AARC Gains |  |  | 2.84 |  |  | 2.85 |  |  | 2.90 |  |  | 2.93 |  |  | 2.90 |

Table S13. Chronological outline of the procedure

| Telephone Screening | |
| --- | --- |
| PAR-Q | 5 Minutes |
| Query Age | 1 Minutes |
|  | = 6 Minutes |
| Procedure of the study on site | |
| Information About the Study | 5 Minutes |
| Consent | 2 Minutes |
| Draw Numbers from 1-100 | 1 Minutes |
| Text Passage | 3 Minutes |
| Measures on Self-perceptions of Aging | 5 Minutes |
| Coordination |  |
| Complex coordination |  |
| Hop-run (up to 2 Attempts) | 2 Minutes |
| Query Difficulty and Exertion | 1 Minutes |
| Ball-grabbing (up to 2 Attempts) | 3 Minutes |
| Query Difficulty and Exertion | 1 Minutes |
| Throw-with-rotation (up to 2 Attempts) | 2 Minutes |
| Query Difficulty and Exertion | 1 Minutes |
| Circles-of-eight (up to 2 Attempts) | 3 Minutes |
| Query Difficulty and Exertion | 1 Minutes |
| Walking Backwards (3 Attempts) | 5 Minutes |
| Query Difficulty and Exertion | 1 Minutes |
| Mobility |  |
| Trunk Bends (2 Attempts) | 2 Minutes |
| Query Difficulty and Exertion | 1 Minutes |
| Shoulder Mobility (2 Attempts) | 3 Minutes |
| Query Difficulty and Exertion | 1 Minutes |
| Strength |  |
| Hand Grip Strength (2 Attempts) | 2 Minutes |
| Query Difficulty and Exertion | 1 Minutes |
| Push-ups | 1 Minutes |
| Query Difficulty and Exertion | 1 Minutes |
| Push-ups | 1 Minutes |
| Query Difficulty and Exertion | 1 Minutes |
| Endurance |  |
| Walking | 20-30 Minutes |
| Query Difficulty and Exertion | 1 Minutes |
| Measures on Sociodemographic Information | 5 Minutes |
| Debriefing | 5-8 Minutes |
| Feedback with Printout of their Results | 3 Minutes |
| Handover of the Five-euro Drugstore (DM) | 1 Minutes |
|  | = 77-90 Minutes |

Table S.14 Exemplar of the printouts

| **Coordination** | | **Points (0-8)** |
| --- | --- | --- |
| Complex Coordination | Hop-run  Ball-grabbing  Throw-with-rotation  Circles-of-eight | 1  1  0  0 |
| Walking Backwards | Speed: 0,42 m/sec | 4 |
| Coordination Score |  | 6:2  = 3  (0-4) |
| **Mobility** | | **Points (0-8)** |
| Trunk Bends | Distance: -1 cm | 2 |
| Shoulder Mobility | Difference: -1 | 0 |
| Mobility Score |  | 2:2  = 1  (0-4) |
| **Strength** | | **Points (0-12)** |
| Hand Grip Strength | Value: 32,1 kg | 4 |
| Push-ups | Quantity: 10/40 sec | 3 |
| Sit-ups | Quantity: 7/30 sec | 2 |
| Strength Score |  | 9:3  = 3  (0-4) |
| **Endurance** | | **Points (0-4)** |
| 2-km-Walking | Time required: 18:10 | 3 |
| **Total Score** | | 10  (0-16) |


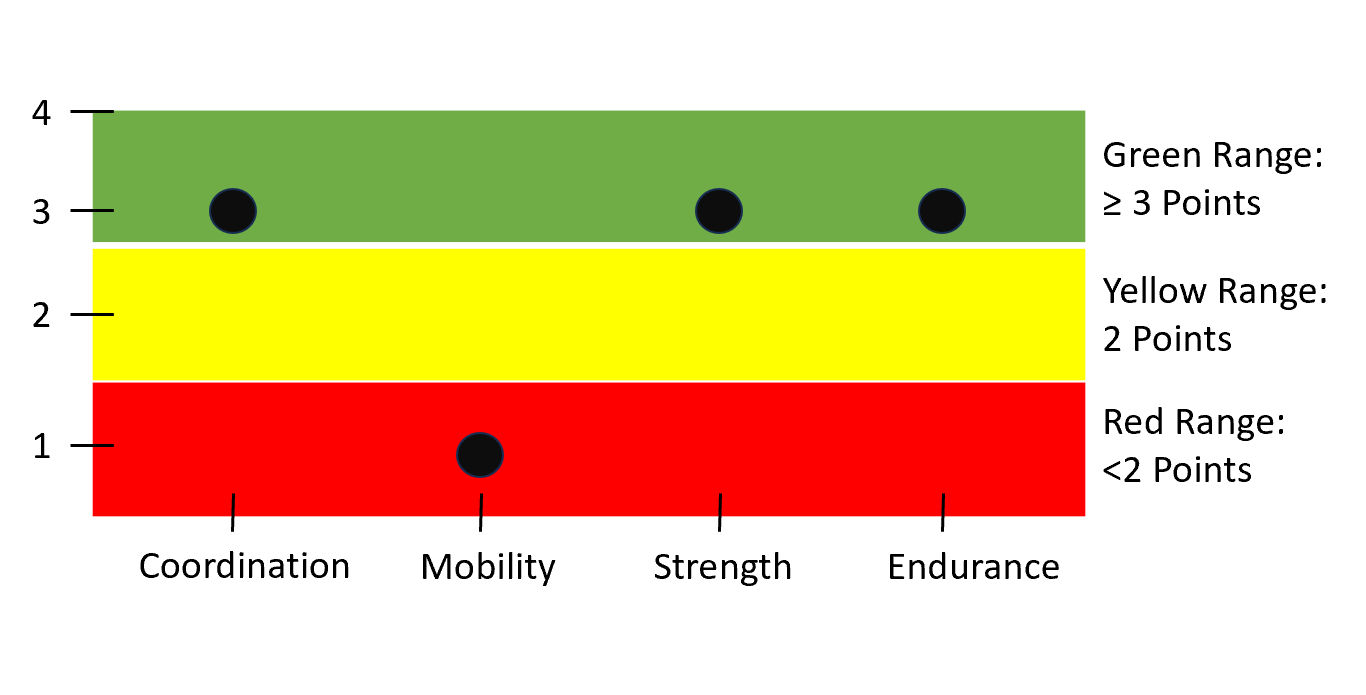


| **Test Results** | **Rating** |
| --- | --- |
| **Heterogeneous Test Profil** | |
| Green-Red | The profile shows strengths and weaknesses. It is advisable to address weaknesses in a targeted manner. |

*from Krell-Rösch et al (2014)
